# Upregulation of *α*3*β*1-Integrin in Podocytes in Early-Stage Diabetic Nephropathy

**DOI:** 10.1155/2016/9265074

**Published:** 2016-06-01

**Authors:** Kaichiro Sawada, Masao Toyoda, Noriko Kaneyama, Sawako Shiraiwa, Hitomi Moriya, Han Miyatake, Eitaro Tanaka, Naoyuki Yamamoto, Masaaki Miyauchi, Moritsugu Kimura, Takehiko Wada, Masafumi Fukagawa

**Affiliations:** Division of Nephrology, Endocrinology and Metabolism, Department of Medicine, Tokai University School of Medicine, 143 Shimokasuya, Isehara, Kanagawa 259-1193, Japan

## Abstract

*Background*. Podocyte injury plays an important role in the onset and progression of diabetic nephropathy (DN). Downregulation of *α*3*β*1-integrin expression in podocytes is thought to be associated with podocyte detachment from the glomerular basement membrane, although the mechanisms remain obscure. To determine the mechanism of podocyte detachment, we analyzed the expression levels of *α*3*β*1-integrin in podocytes in early and advanced stages of DN.* Methods*. Surgical specimens from DN patients were examined by* in situ* hybridization, and the expression levels of *α*3- and *β*1-integrin subunits in glomeruli of early (*n* = 6) and advanced (*n* = 8) stages were compared with those of normal glomeruli (*n* = 5). Heat-sensitive mouse podocytes (HSMP) were cultured with TGF-*β*1 to reproduce the microenvironment of glomeruli of DN, and the expression levels of integrin subunits and the properties of migration and attachment were examined.* Results*. Podocytes of early-stage DN showed upregulation of *α*3- and *β*1-integrin expression while those of advanced stage showed downregulation. Real-time PCR indicated a tendency for upregulation of *α*3- and *β*1-integrin in HSMP cultured with TGF-*β*1. TGF-*β*1-stimulated HSMP also showed enhanced* in vitro* migration and attachment on collagen substrate.* Conclusions*. The results suggested that podocyte detachment during early stage of DN is mediated through upregulation of *α*3*β*1-integrin.

## 1. Introduction

Diabetic nephropathy (DN) is one of the major indications for hemodialysis treatment in patients with diabetes mellitus (DM). It is important to elucidate the cause of DN and develop more effective treatments based on the escalating medical costs and need for improvement of quality of life (QOL) of DN patients. Previous studies showed that the onset and progression of DN correlate with injury of glomerular epithelial cells (podocytes) [1–7]. Furthermore, effacement of foot process followed by podocyte detachment from the glomerular basement membrane (GBM) is known to result in proteinuria [8]. Among the many molecules that are known to be involved in cell-to-cell and cell-to-substrate attachment in glomeruli, *α*3*β*1-integrin is expressed primarily in podocytes and considered to play a critical role in the attachment of podocytes to the GBM [9, 10]. Several studies have so far examined changes in *α*3*β*1-integrin expression in podocytes in patients with various types of nephropathies and equivalent animal model [11–19]. The results of studies involving DN patients, animal models of DN, and* in vitro* culture of podocytes under high glucose conditions have so far demonstrated underexpression of *α*3-integrin subunit in podocytes and suggested that the main cause of podocyte detachment was a decrease in the number of *α*3*β*1-integrins on the surface of podocytes [20–23]. However, the mechanism responsible for the downregulation of *α*3*β*1-integrin during progression of DN remains obscure.

TGF-*β*1 is synthesized and secreted by glomerular mesangial cells (MC) in patients with nephropathy and animal models of glomerulonephritis [24]. The secreted TGF-*β*1 from MC stimulates podocyte to synthesize collagen type IV [25, 26]. Furthermore, TGF-*β*1 is considered to be one of the most important factors involved in the reproduction of microenvironment of DN glomeruli in culture systems [27, 28], though almost all* in vitro* studies have been performed using podocytes cultured under high glucose conditions.

The present study was designed to determine the mechanism of podocyte detachment in DN. For this purpose, we examined the expression of *α*3- and *β*1-integrin subunits in glomeruli of patients with early and advanced stages of DN. We also reproduced the changes, including changes in the expression levels of *α*3*β*1-integrin in an* in vitro* culture of heat-sensitive mouse podocytes (HSMP). Contrary to the data of previous studies [20–23], the results showed upregulation of *α*3*β*1-integrin expression in podocytes in early-stage DN. The results were confirmed in* in vitro* culture of TGF-*β*1-stimulated mouse podocytes.

## 2. Methods

### 2.1. Subjects

Renal biopsy tissues of 14 patients with DN were used. The diagnosis of DN was confirmed by histopathological examination and the severity was classified into two grades: grade I (DN1 = 6 patients) reflected mild mesangial expansion and DN grade II (DN2 = 8 patients) represented moderate mesangial expansion. Control samples were obtained from uninvolved portions of surgically removed kidneys afflicted with malignancies (5 patients). After resection, the samples were embedded in optimal cutting temperature (OCT) compound (Tissue Tek; Miles, Elkhart, IN) and stored at −70°C until use. Blood and urine samples were collected from subjects immediately before renal biopsy. Serum creatinine, total protein, HbA1c, urinary protein, and creatinine clearance were measured using standard methods in our hospital (Table 1).

This study was reviewed and approved by the Institutional Review Board of Tokai University School of Medicine. All procedures were performed in accordance with the principles of the Declaration of Helsinki. Informed consents were obtained from all subjects.

### 2.2. *In Situ* Hybridization

Oligonucleotide probes for mRNAs of human *α*3- and *β*1-integrin subunits were labeled using a digoxigenin (DIG) oligonucleotide tailing kit according to the standard protocol (Boehringer Mannheim, Mannheim, Germany). Free DIG was removed by ethanol precipitation and probes were dissolved in diethylpyrocarbonate-treated water.

*In situ* hybridization was performed according to a protocol developed in our laboratory with minor modifications. Briefly, sections of specimens were fixed in 4% paraformaldehyde in phosphate-buffered saline (PBS) and then deproteinized with HCl and digested with proteinase K (Sigma Chemical, St. Louis, MO). Following a treatment with a hybridization buffer, specimens were hybridized overnight with a DIG-labeled oligonucleotide probe in the same buffer. After washing with a stringent condition, the DIG-labeled probe was detected immunohistochemically using a mouse monoclonal anti-DIG antibody (Boehringer Mannheim), horseradish peroxidase- (HRP-) conjugated rabbit anti-mouse IgG antibody (Dako, Glostrup, Denmark), and HRP-conjugated swine anti-rabbit IgG antibody (Dako). The reaction was visualized with diaminobenzidine tetrahydrochloride in 0.05 M Tris-HCl, pH 7.6, and 0.03% H_2_O_2_. Sections were briefly counterstained with hematoxylin, rinsed, dehydrated, cleared in xylene, and mounted. Three independent investigators who were blinded to the results of histopathological classification counted the numbers of stained cells. The percentage of mRNA-positive cells relative to the total glomerular cells was determined.

### 2.3. Cell Culture

HSMP were cultured with RPMI 1640 medium (Nissui Pharma, Tokyo, Japan) containing 10% fetal bovine serum, 50 U/mL penicillin, and 50 mg/mL streptomycin in 5% CO_2_-95% air atmosphere. Cells proliferated at 33°C in the presence of 50 units/mL mouse recombinant IFN-*γ* (Aviva System Biology, San Diego, CA). For podocyte differentiation, cells between passages 10 and 13 were cultured at 37°C without IFN-*γ* for two weeks. Podocyte differentiation was confirmed by the expression of synaptopodin detected by quantitative real-time PCR. To set the conditions of microenvironment to mimic those of DN glomeruli, 1 ng/mL TGF-*β*1 (R&D Systems, Minneapolis, MN) was added to medium. The medium was replaced with a new one every other day.

### 2.4. Attachment and Migration Assays

HSMP were differentiated as above, and 1 ng/mL TGF-*β*1 was added before three days of experiments. Cells were harvested by trypsinization, counted, and inoculated into plates as described below.

To measure the ability of attachment of differentiated HSMP to the substrate, 1 × 10^4^ cells per well were inoculated into 24-well plate coated with collagen type IV and incubated at 37°C. After 30-minute incubation, the plates were shaken to detach weakly binding cells, washed several times with PBS(−), refilled with culture medium, and incubated at 37°C in 5% CO_2_. The next day the cells were fixed with 4% paraformaldehyde and photographed. The number of cells in the view fields was counted.

To measure the ability of differentiated HSMP to migrate towards the substrate, the Oris Pro cell migration assay kit (Platypus Technology, Fitchburg, WI) was used. 4 × 10^4^ cells per well were inoculated into 96-well plate coated with collagen type I supplied with the kit. After 24 hours, the cells were fixed with 4% paraformaldehyde and stained with hematoxylin. Absorbance of 600 nm light passed into the central detection zone was measured by spectrophotometry (Infinite F200PRO, TECAN, Männedorf, Switzerland).

### 2.5. Quantitative Real-Time Polymerase Chain Reaction

Differentiated HSMP were inoculated into wells of 48-well plate coated with collagen type IV with a rate of 1 × 10^6^ per well and cultured with 1 ng/mL TGF-*β*1 at 37°C under 5% CO_2_-95% air atmosphere. According to the time schedule, total RNAs were recovered from cells using the RNAqueous RNA purification kit (ThermoFisher, Waltham, MA) and converted to cDNA using SuperScript III First-Strand Synthesis System (ThermoFisher). Quantitative real-time polymerase chain reaction (qRT-PCR) was performed with the cDNA, as described previously [29]. The reaction mixture was prepared as described in the instruction manual of the kit (TaqMan Gene Expression Assays; ThermoFisher) that also contained the primers and probes for mouse *α*3-integrin (assay ID: Mm00442910_m1), *β*1-integrin (assay ID: Mm01253230_m1), and 18S ribosomal RNA (rRNA), which was used as the endogenous control. PCR was performed on an ABI PRISM 7500 (ThermoFisher). Data were analyzed by the comparative Ct method, and the amounts of integrin mRNAs were expressed relative to that of 18S rRNA.

### 2.6. Statistical Analysis

Results are expressed as mean ± SD. Differences between two groups were assessed by Student's *t*-test. Spearman's correlation coefficient analysis was used to examine the relationship between parameters. *p* < 0.05 was considered statistically significant.

## 3. Results

### 3.1. Patient Characteristics

The clinical characteristics of the patients are shown in Table 1. Based on the degree of mesangial expansion, patients were categorized into the DN1 and DN2 groups, as described in Methods. Serum creatinine, urinary protein, and creatinine clearance worsened significantly during the course of DN in DN2 patients compared with DN1 patients.

### 3.2. Expression of Integrin Subunits Detected by* In Situ* Hybridization

**Figure 1:**
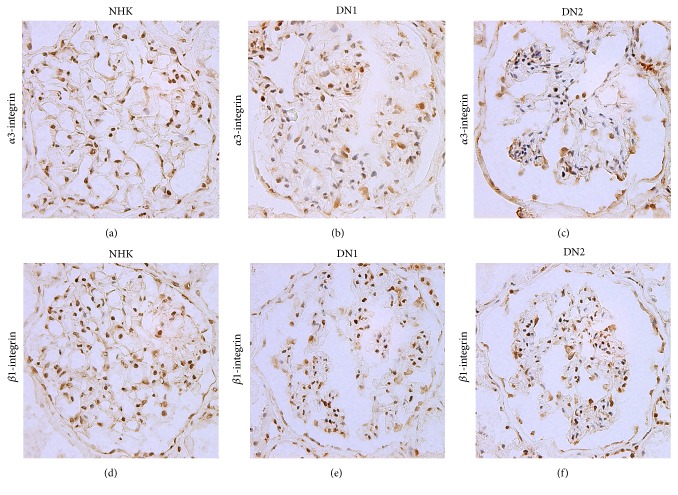
*In situ* hybridization of *α*3- and *β*1-integrin subunits. Representative mRNA expression of integrin subunits in glomeruli of normal human kidney, early (DN1) and advanced (DN2) stages of DN. Integrin expression is colored by DAB (brown), and nuclei of cells are stained by hematoxylin (blue) (magnification, ×100).

**Figure 2:**
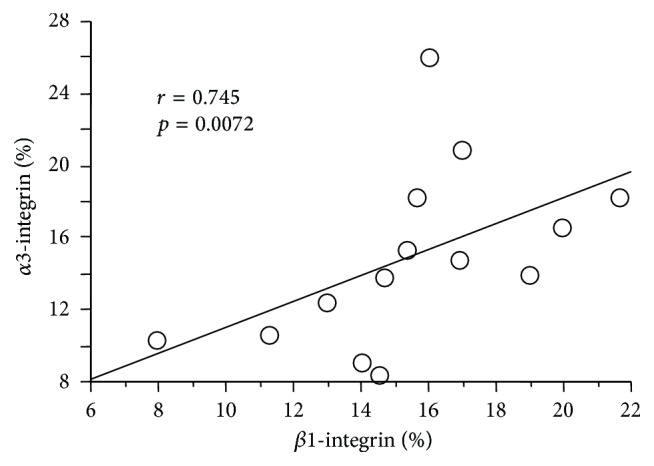
Strong correlation between the expression levels of *α*3- and *β*1-integrin subunits in DN glomeruli of 14 DN patients. The percentages of cells positive for *α*3- and *β*1-integrin in glomeruli were examined by* in situ* hybridization.

Representative glomeruli stained for *α*3- and *β*1-integrin are shown in Figure 1 and the percentages of cells positive for these mRNAs are summarized in Table 2. Although the percentage of cells positive for each integrin subunit in glomeruli was not significantly different to those of NHK when data of all DN patients were analyzed, those of DN1 were significantly higher (Table 2). Particularly, the percentage of cells positive for *β*1-integrin mRNA was significantly higher in DN1 than NHK. Furthermore, the percentages of cells positive for *α*3- and *β*1-integrin subunits were both significantly lower in DN2. Further analysis showed strong correlation between percentages of cells positive for *α*3- and *β*1-integrin subunits (Figure 2), suggesting that the increase in the subunits resulted in the formation of *α*3*β*1-integrin on the podocyte surface.

### 3.3. Induction of *α*3- and *β*1-Integrin Expression in Cultured Mouse Podocytes

**Figure 3:**
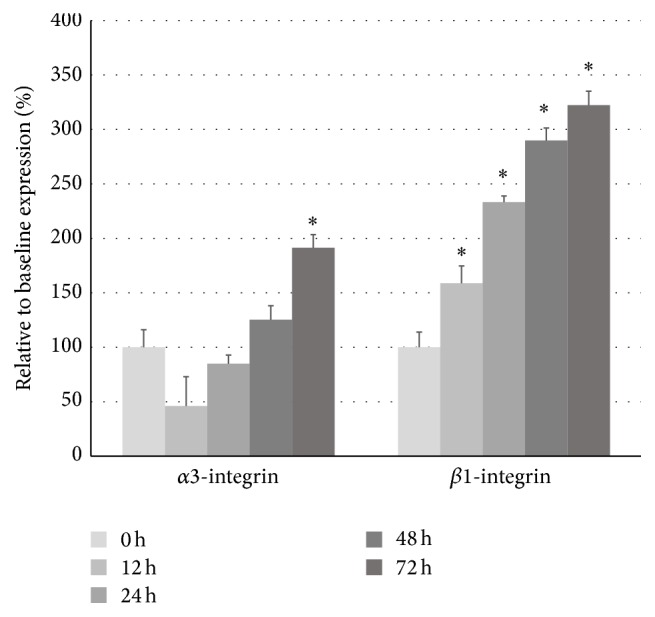
Expression levels of *α*3- and *β*1-integrin subunits in cultured mouse podocytes (HSMP) treated with TGF-*β*1. Differentiated HSMP were cultured with 1 ng/mL TGF-*β*1, and the expression levels of *α*3- and *β*1-integrin were measured by semiquantitative real-time PCR. The relative mRNA level at each time point is represented as a percentage of the baseline value (0 hours). ^*∗*^ *p* < 0.05 versus the baseline value.

Next, we induced the expression of integrin subunits in DN1 glomeruli in cultured HSMP. To mimic the condition of DN glomerular microenvironment, the cultured cells were treated with 1 ng/mL TGF-*β*1 for three days and RNAs were recovered at various time points. Although the expression of *α*3-integrin gene decreased significantly at the onset of the experiment, it recovered after 48 hours of culture and finally reached about twice the expression level at baseline (0 hours) (Figure 3). On the other hand, the expression of *β*1-integrin gene increased steadily and reached more than three times the baseline expression level at 72 hrs (Figure 3). Thus, the expression levels of both integrins increased proportionally with culture duration in the presence of TGF-*β*1. These results demonstrated that induction of *α*3- and *β*1-integrin subunits in glomeruli of DN1 patients can be reproduced by HSMP cultured with TGF-*β*1. On the other hand, enhanced detachment of cells from substrate was not observed during the experiments, and TGF-*β*1 did not affect the number of cells under these conditions (data not shown).

### 3.4. TGF-*β*1 Enhances Substrate Attachment and Migration of HSMP

**Figure 4:**
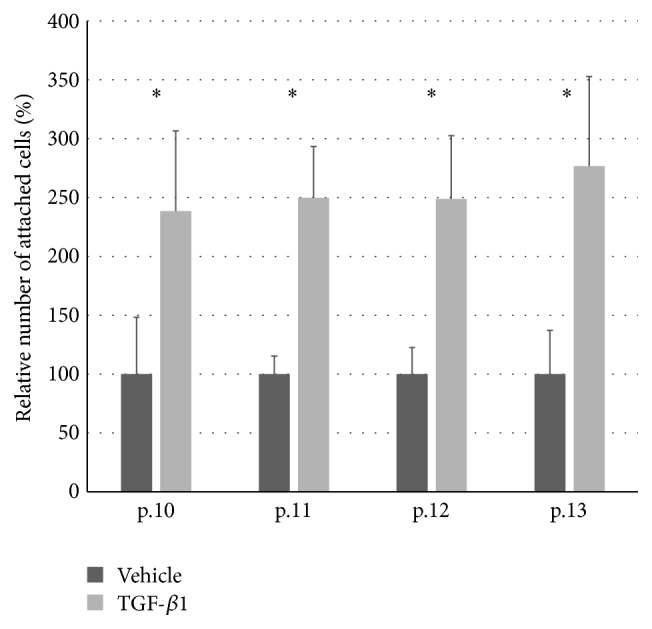
Attachment assay of HSMP cultured with/without TGF-*β*1. Attachment assay was performed as described in Methods using cells of passages 10–13. The *y*-axis shows percentage of attached HSMP cultured with TGF-*β*1 relative to that of HSMP cultured with the vehicle. ^*∗*^ *p* < 0.05 versus vehicle.

**Figure 5:**
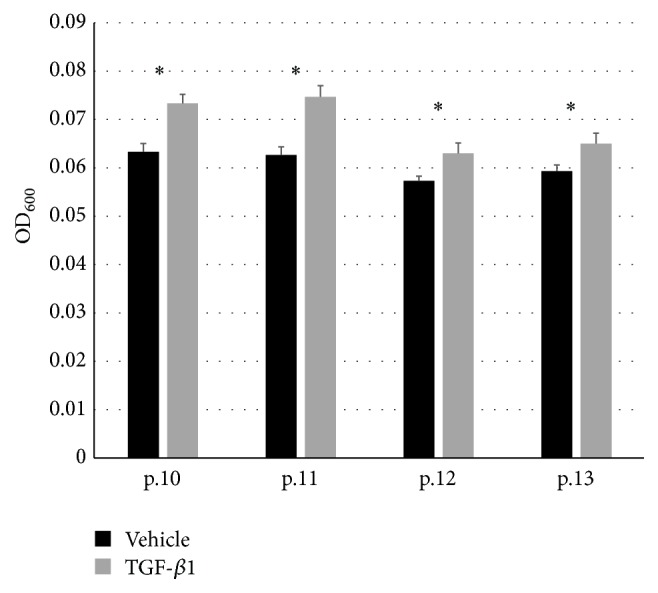
Migration assay of HSMP cultured with/without TGF-*β*1. Migration assay was performed as described in Methods using cells of passages 10–13. Absorbance at 600 nm (OD_600_) of migrated HSMP stained with hematoxylin. ^*∗*^ *p* < 0.05 versus vehicle.

Finally, we examined the effects of TGF-*β*1-induced expression of *α*3*β*1-integrin on substrate attachment and cell migration. For substrate attachment, type IV collagen-coated plates were shaken and washed after 30 minutes of cell inoculation, and the number of remaining cells was counted in the presence or absence of TGF-*β*1. On the other hand, migration was measured by using a cell migration assay kit, in which the extent of migration was measured by absorbance of stained cells that entered the central clear space of the plates. TGF-*β*1 significantly enhanced HSMP attachment to type IV collagen (Figure 4) and their migration on type I collagen-coated plate (Figure 5). These results suggest the involvement of both processes in the increased expression of *α*3*β*1-integrin induced by TGF-*β*1.

## 4. Discussion

The main finding of the present study was upregulation of integrin expression in podocytes of patients with early DN and in HSMP cultured with TGF-*β*1.

Since no proliferation of mature podocytes is noted in the majority of nephropathies, including DN [30], integrin mRNA overexpression in podocytes, including higher percentage of integrin-positive cells in DN1 glomeruli, can be detected by* in situ* hybridization. Although several studies involving DN patients and animal models reported a decrease or no change in *α*3*β*1-integrin expression in podocytes, almost all of them assessed the expression of integrin in podocytes of proceeded stage of DN in patients and animals [20–23]. The present study also showed no significant change in *α*3- and *β*1-integrin expression when data of all DN patients were pooled together and compared with those of NHK (Table 2). On the other hand, separate analysis of data of patients with early stage (DN1) demonstrated significant upregulation of integrin, which could have been masked in previous studies. Interestingly, controversial results regarding the expression patterns of integrin in podocytes have been reported in various types of nephropathies and glomerulonephritis other than DN [11–19]. In general, proteinuria correlates with downregulation of integrin in podocytes in various nephropathies, although an increase or no change in integrin expression has been reported in some cases of early-stage nephropathy. For example, overexpression of glomerular *α*3-integrin was reported in a rat model of minimal-change nephropathy 10 days after injection of puromycin aminonucleoside (PAN) [17]. Furthermore, Baraldi et al. reported downregulation of *α*3-integrin in 6 patients with stage I-III membranous nephropathy [11], whereas Bains et al. reported little change in integrin expression in 18 patients with “early-” stage proteinuria [16] and Chen et al. used a rat model of streptozotocin-induced DN to report no change in *α*3-integrin expression within the first week followed by a decrease after 1–3 months [21].

The upregulated expression of integrin in DN1 podocytes probably represents increased turnover and reconstruction of adhesion structures responsible for cell-substrate interaction [16]. This argument is supported by the finding of enhanced attachment and migration of HSMP with upregulated integrin expression. The reconstruction of adhesion structures could be a mechanism to counteract increased instability of DN1 podocytes caused by the effacement of foot process.

In the present study, HSMP were cultured with TGF-*β*1 instead of high glucose concentrations to reproduce the microenvironment in DN glomeruli. Previous studies reported that high glucose concentrations in culture medium result in downregulation of *α*3-integrin expression in podocytes within 24 hours, although the induction of *α*3-integrin as observed in DN1 podocytes has not been reported [21, 22]. It is likely that hyperglycemia does not directly change integrin expression in the early stages of DN. On the other hand, TGF-*β*1 is produced abundantly in mesangial proliferative glomerulonephritis [24, 27, 28] and induces podocytes to synthesize collagen type IV, which contributes to the proliferation of GBM [25, 26]. Moreover, TGF-*β*1 induces MC to synthesize type I collagen and *β*1-integrin, which contribute to the proliferation of mesangial substrates [31]. These evidences suggest that TGF-*β*1, rather than hyperglycemia, is directly involved in the regulation of cell-substrate interaction of podocytes and MC in glomeruli.

TGF-*β*1 is also known to induce cell death in certain types of cells [32, 33]. Our results showed death of HSMP after their detachment from the substrate when they were cultured with TGF-*β*1 at concentrations higher than that used in experiments of the present study (data not shown). Although upregulation of genes involved in cell death of podocytes cultured under high glucose concentrations was reported [34], some studies described maintaining cultured podocytes under high glucose concentrations as long as 1-2 weeks and even more than 6 months [35, 36]. These studies suggest that direct stimulation by high glucose concentration may not be sufficient to induce podocyte detachment. One can probably establish a culture system that allows the process of detachment and death of podocytes.

In conclusion, upregulation of *α*3*β*1-integrin expression was detected in early-stage DN, suggesting reconstruction of adhesion structures essential for cell-substrate interaction after effacement of foot process. HSMP cultured with TGF-*β*1 reproduced *α*3- and *β*1-integrin upregulation observed in DN1, and podocytes stimulated with TGF-*β*1 showed enhanced attachment and migration. Our* in vitro* culture system that mimics the onset and progression of DN can be used to elucidate the mechanism of podocyte detachment and to develop effective treatments for proteinuria.

## Figures and Tables

**Table 1:** Baseline characteristics.

	NHK (*n* = 5)	DN1 (*n* = 6)	DN2 (*n* = 8)
Gender (M/F)	4/1	4/2	6/2
Age (years)	45 ± 10	44 ± 17	48 ± 12
Serum creatinine (mg/dL)	0.9 ± 0.2	0.7 ± 0.1	1.1 ± 0.3^*∗*^
Total protein (g/dL)	7.4 ± 0.8	6.9 ± 0.6	6.0 ± 1.6
HbA1c (%)	ND	8.7 ± 2.3	8.8 ± 2.9
Urinary protein (g/day)	ND	0.22 ± 0.15	1.95 ± 2.45^*∗*^
Creatinine clearance (mL/min)	ND	97.0 ± 20.5	73.3 ± 26.0^*∗*^

Data are expressed as mean ± SD.

^*∗*^ *p* < 0.05 versus DN1.

ND: not determined.

**Table 2:** Percentages of cells positive for integrin mRNAs in glomeruli.

	NHK (*n* = 5)	Patients with diabetic nephropathy		
		Total (*n* = 14)	DN1 (*n* = 6)	DN2 (*n* = 8)
*α*3-integrin (%)	14.1 ± 2.8	14.9 ± 4.9	17.9 ± 4.4	12.0 ± 3.5^*∗*^
*β*1-integrin (%)	12.6 ± 4.4	15.5 ± 3.5	17.6 ± 2.6	13.3 ± 3.0^¶^

Data are expressed as mean ± SD.

^*∗*^ *p* < 0.05 versus DN1.

^¶^ *p* < 0.01 versus DN1.
